# Mindful eating and Chrono-Mediterranean diet adherence associated with lower gastrointestinal symptom burden in young adults

**DOI:** 10.3389/fmed.2026.1835659

**Published:** 2026-06-23

**Authors:** Canan Altinsoy, Yagmur Demirel Ozbek, Isa Celik

**Affiliations:** 1 Department of Nutrition and Dietetics, Faculty of Health Sciences, Recep Tayyip Erdoğan University, Rize, Türkiye; 2 Department of Child Health and Disease Nursing, Faculty of Health Sciences, Recep Tayyip Erdoğan University, Rize, Türkiye

**Keywords:** eating attitude, gastrointestinal health, gastrointestinal symptoms, gut-brain axis, Mediterranean diet, mindful eating

## Abstract

**Objectives:**

This study aimed to investigate the relationship between gastrointestinal symptom burden and Chrono-Mediterranean diet adherence and mindful eating in young adults. It also sought to explore the role of microbiota awareness in this relationship and identify factors associated with Chrono-Mediterranean diet adherence.

**Methods:**

This cross-sectional study involved 677 young adults and employed convenience and snowball sampling. Data were collected through face-to-face interactions using a personal information form, the Gastrointestinal Symptom Rating Scale (GSRS), the Bristol Stool Chart, the Chrono-Mediterranean Diet Score (CMDS), the Expanded Mindful Eating Scale (EMES), and the Microbiota Awareness Scale (MAS). Descriptive statistics, correlation analyses, and multiple linear regression analyses were performed using SPSS (IBM, NY, USA) version 30.0, with statistical significance set at *p* < 0.05.

**Results:**

Among the 677 respondents (mean age: 20.79 ± 2.34 years; 75.2% female), GSRS scores were negatively correlated with microbiota awareness (*r* = −0.182, *p* < 0.001) and the CMDS (*r* = −0.141, *p* < 0.001), but were not correlated with mindful eating. However, microbiota awareness was positively correlated with mindful eating (*r* = 0.092, *p* = 0.017) and the CMDS (*r* = 0.205, *p* < 0.001). In multivariable analyses, male sex, non-smoking, higher levels of mindful eating, and higher CMDS scores independently predicted a lower gastrointestinal symptom burden (*R*^2^ = 0.11, *p* < 0.001). Higher CMDS scores were independently predicted by physical activity, not smoking, not drinking, increased microbiota awareness, and higher levels of mindful eating, whereas obesity was identified as a negative predictor (*R*^2^ = 0.15, *p* < 0.001).

**Conclusion:**

Greater adherence to the Chrono-Mediterranean diet and higher levels of mindful eating were independently associated with lower gastrointestinal symptom burden in young adults. Although microbiota awareness was associated with symptom burden in univariate analyses, it was not an independent predictor after adjustment. These findings highlight the potential relevance of diet quality, meal timing-related dietary adherence, and eating-related awareness for gastrointestinal health in young adults.

## 1 Introduction

Gastrointestinal health plays an important role in overall health, as it affects immune regulation and metabolic homeostasis (1). Gastrointestinal symptoms, such as abdominal pain, bloating, constipation, and diarrhea, can substantially impair an individual’s quality of life (2). These symptoms cause physical discomfort and may interfere with daily functioning, productivity, and psychosocial well-being, highlighting their relevance beyond the gastrointestinal system (3–5). Evidence suggests that genetic factors, psychosocial status, dietary habits, physical activity, lifestyle characteristics, and gut microbiota contribute to the development of gastrointestinal symptoms (6, 7). As gastrointestinal symptoms are increasingly conceptualized within the framework of gut–brain interaction disorders, identifying modifiable dietary and behavioral factors associated with symptom burden may also be relevant to psychosocial well-being.

The Mediterranean diet is one of the most extensively studied dietary patterns associated with gastrointestinal health (8, 9). This diet is characterized by high consumption of vegetables, fruits, whole grains, legumes, olive oil, and fish. It is also rich in fiber, polyphenols, and other bioactive compounds (8, 9). This dietary pattern is associated with reduced inflammation, improved metabolic health, and increased diversity of gut microbiota. Recent studies have shown that individuals who adhere closely to the Mediterranean diet experience improved gastrointestinal function and reduced digestive symptoms (10, 11). In addition, growing evidence suggests that the timing of food intake may be as important as food composition in shaping gastrointestinal health (12, 13). The concept of food intake timing, known as chrononutrition, is a research field that examines the interactions between eating patterns and metabolism in harmony with biological rhythms (14).

Biological rhythms regulate several physiological processes, including gastrointestinal motility, hormone secretion, digestive enzyme activity, and gut microbiota dynamics (15, 16). Misalignment between food intake timing and biological rhythms may contribute to metabolic disturbances and impaired gastrointestinal function (17, 18). In response to this, the Chrono-Mediterranean diet approach has emerged, which combines the principles of the Mediterranean diet with chrononutrition to create a holistic dietary model. In this diet model, in addition to the principles of the Mediterranean diet, the timing of food consumption is aligned with biological rhythms (19). It has been suggested that adherence to the Chrono-Mediterranean diet may have potential beneficial effects on the regulation of metabolic function, inflammation, and gut microbiota (20, 21).

In addition to dietary patterns, eating behaviors may also play an important role in gastrointestinal health (22). One related construct that has gained increasing attention is mindful eating, which involves a conscious approach to food consumption characterized by awareness of hunger and satiety cues (23). Higher levels of mindful eating not only influence dietary choices but may also promote healthier eating patterns and potentially reduce the risk of gastrointestinal disturbances (24). Another concept that is increasingly important for gut health is microbiota awareness. The level of knowledge and awareness individuals have about the microbiota has become an intriguing area of research, particularly as the significant role of gut microbiota in human health has become better understood (25, 26). Greater microbiota awareness may encourage the adoption of dietary behaviors that support gut health (26).

Previous studies have examined the health-related roles of the Mediterranean diet, chrononutrition, mindful eating-related constructs, and microbiota-related factors (11, 17, 24, 26). However, few studies have evaluated these factors simultaneously in relation to gastrointestinal symptoms, particularly in young adults. To the best of our knowledge, no previous study has jointly examined mindful eating, microbiota awareness, and Chrono-Mediterranean diet adherence within the framework of gut–brain interactions. However, the direction and magnitude of these relationships in this population remain to be elucidated. Therefore, this study aims to examine the associations between gastrointestinal symptom burden and Chrono-Mediterranean diet adherence, mindful eating, and microbiota awareness in young adults. Microbiota awareness is included as a complementary construct that reflects perceived, rather than objectively measured, characteristics of the gut microbiota. It is hypothesized that greater Chrono-Mediterranean diet adherence, higher levels of microbiota awareness, and higher levels of mindful eating will each be negatively associated with gastrointestinal symptom burden, while also being positively associated with one another.

## 2 Methods

### 2.1 Study population and sample

The sample size for this study was calculated using G*Power 3.1.9.7 (Heinrich Heine University, Düsseldorf, Germany). For the multiple linear regression analysis, the sample size was calculated assuming an effect size of f^2^ = 0.20, a margin of error of 5% (*α* = 0.05), and a statistical power of 95% (1 − *β* = 0.95), resulting in a minimum required sample size of 465 individuals. To account for potential data loss, the sample size was increased by 10%, leading to a target of at least 520 participants in the study (27, 28). The study ultimately included 677 participants.

Young adults aged 18–30 were included in the study, and informed consent was obtained from all participants. To be included in the study, participants were required to sign a consent form and complete a questionnaire. Questionnaires with incomplete responses or incorrect answers were excluded, and individuals with chronic gastrointestinal illnesses and those with cognitive-communication problems were also not included in the study.

### 2.2 Data collection

A face-to-face survey method was used to collect the research data. A random sampling method was employed to recruit the participants, and the snowball sampling technique was used to expand the sample size with the help of the participants. Ethical approval for this study was obtained from the Social and Human Sciences Ethics Committee of Recep Tayyip Erdoğan University (Decision No: 2025/709 dated 11 December 2025). The research was conducted in accordance with the principles of the Declaration of Helsinki. Participants were informed about the purpose of the study and the duration of the survey, and their consent was obtained. The research questionnaire was prepared by the authors and included the following: Personal Information, Gastrointestinal Symptom Rating Scale (GSRS), Chrono-Mediterranean Diet Score (CMDS), Expanded Mindful Eating Scale (EMES), and Microbiota Awareness Scale (MAS).

### 2.3 Data collection tools

#### 2.3.1 Personal information form

In the study, a Personal Information Form created by the researchers was used to determine the sociodemographic characteristics and lifestyle of the participants; it included questions about the participants’ age, sex, marital status, smoking habits, alcohol consumption, physical activity level, and sleep duration. All items in the personal information form were open-ended, allowing participants to report their responses in their own words. Additionally, the participants’ body weight (kg) and height (cm) were measured by the researchers, and the Body Mass Index (BMI) was calculated by dividing body weight (kg) by the square of height (m) (kg/m^2^) (29).

#### 2.3.2 Gastrointestinal symptom rating scale (GSRS)

The Gastrointestinal Symptom Rating Scale (GSRS) was developed by Revicki et al. to identify individuals’ experiences with gastrointestinal symptoms and assess common symptoms observed in gastrointestinal diseases (30). It consists of 15 items. The items of the scale are evaluated using a seven-point Likert-type scale, ranging from ‘no problem’ to ‘very severe discomfort.’ The scale comprises five subdomains: diarrhea, indigestion (dyspepsia), constipation, abdominal pain, and reflux. Participants were asked to evaluate the gastrointestinal symptoms they experienced over the past week. Higher scores on the scale indicate greater severity of gastrointestinal symptoms. The Turkish validity and reliability study of the scale was conducted by Turan et al., who reported a Cronbach’s alpha of 0.82 (31). In the current study, the Cronbach’s alpha for the scale was 0.89.

#### 2.3.3 Bristol stool chart

The Bristol stool chart was used to categorize the consistency and shape of human feces. Scores of 1–2 indicate constipation, 3–5 are considered normal, and scores of 6 or 7 are classified as diarrhea (32, 33).

#### 2.3.4 Chrono-Mediterranean diet score (CMDS)

The Chrono-Mediterranean Diet Score (CMDS) was developed by De Matteis et al. to assess individuals’ eating habits based not only on adherence to the Mediterranean diet but also by considering the principles of chrononutrition (19). The CMDS evaluates individuals’ daily and weekly food consumption frequencies of 11 food categories: fruits, vegetables, legumes, baked goods (e.g., bread, pasta, biscuits), cereal products, fish, meat and meat products, milk and dairy products, olive oil, butter/margarine/animal fats, and alcohol. When developing the CMDS, factors related to chrononutrition, such as the timing of food consumption and physical activity levels, along with the core components of the Mediterranean diet, were also included in the assessment. The total score obtained from the scale ranges from −13 to 25, with higher scores indicating a greater adherence to the Chrono-Mediterranean diet (19). A validity and reliability study of the scale in Turkish was conducted by Koçak et al. (21).

#### 2.3.5 Expanded mindful eating scale (EMES)

The Expanded Mindful Eating Scale (EMES) was developed by Kawasaki et al. to assess eating behaviors that focus on the food consumed without being influenced by environmental factors and without judging food choices by individuals internalizing the concepts of physical hunger and satiety, along with awareness of the effects of emotions and thoughts (34). The scale consists of 20 items and five sub-dimensions (planetary health, awareness and appreciation of food, reactivity, non-judgmental awareness, and hunger and satiety signals). The items are scored using a four-point Likert-type scale evaluation system, ranging from 1 (strongly disagree) to 4 (strongly agree). Higher scores on this scale indicate higher levels of mindful eating (34). A validity and reliability study of the scale in Turkish was conducted by Doğan et al. (35). In the Turkish validation study, the Cronbach’s alpha for the EMES was reported as 0.64 (35). In the current study, the Cronbach’s alpha for the scale was found to be 0.60.

#### 2.3.6 Microbiota awareness scale (MAS)

The Microbiota Awareness Scale (MAS) developed by Külcü and Önal was used to assess microbiota awareness (25). The scale consists of 20 items and four sub-dimensions (general knowledge, product knowledge, chronic diseases, and probiotic–prebiotic awareness). The items on the scale are evaluated using a five-point Likert-type scale scoring system ranging from 1 (strongly disagree) to 5 (strongly agree). The lowest possible score on the scale is 20, whereas the highest is 100. There is no cut-off point on the scale; an increase in the score indicates a higher level of microbiota awareness in the individual (25). In the original study conducted by the developers of the scale, the Cronbach’s alpha was reported as 0.85 (25). In the current study, the Cronbach’s alpha for the scale was found to be 0.86.

### 2.4 Statistical analysis

Statistical analysis was conducted using the SPSS 30.0 software package. Descriptive statistics for continuous variables are presented as means, standard deviations, and minimum and maximum values; descriptive statistics for categorical variables are expressed as frequencies and percentages. The suitability of the normal distribution for continuous variables was assessed by examining skewness and kurtosis values. The skewness and kurtosis values for all scales were within the range of −2 to +2. Pearson’s correlation analysis was used to examine the relationships between the variables. Multiple linear regression analysis was performed to identify the factors affecting the Chrono-Mediterranean Diet Score and the GSRS. Separate multiple linear regression models were constructed with the GSRS and the Chrono-Mediterranean Diet Score as dependent variables. Variables that were considered relevant to the study aims and conceptually associated with the outcomes were simultaneously entered into the model. This approach was used to assess the independent contribution of each variable while accounting for potential confounding effects. Before the regression analyses, multicollinearity among the independent variables was assessed by calculating the variance inflation factor (VIF) and tolerance values. All VIF values were below 10, and all tolerance values were above 0.10, indicating no evidence of problematic multicollinearity in the regression models. A significance level of *p* < 0.05 was accepted for statistical analyses.

## 3 Results

A total of 677 participants were included in this study. The mean age of the participants was 20.79 years (SD = 2.34), ranging from 18 to 30 years old. The average sleep duration was 7.40 h (SD = 1.19), with values ranging from 4 to 13 h. The mean body mass index (BMI) was 23.18 kg/m^2^ (SD = 4.05), with a range between 15.94 and 45.79 kg/m^2^. In terms of sex distribution, the majority of participants were women (75.2%, *n* = 509), while 24.8% (*n* = 168) were men. Regarding smoking status, 20.7% (*n* = 140) of the participants reported being smokers, whereas 79.3% (*n* = 537) were non-smokers. Similarly, 7.7% (*n* = 52) of the participants reported consuming alcohol, while the majority (92.3%, *n* = 625) indicated that they did not. With respect to physical activity, 27.3% (*n* = 185) of the participants reported being physically active, whereas 72.7% (*n* = 492) were inactive. Examination of Bristol stool types showed that the majority of participants had normal stool consistency (85.4%, *n* = 578), whereas 8.7% (*n* = 59) reported being constipated and 5.9% (*n* = 40) reported having diarrhea. According to BMI classifications, 64.8% (*n* = 439) of the participants had a normal body weight, 20.7% (*n* = 140) were overweight, 8.7% (*n* = 59) were underweight, and 5.8% (*n* = 39) were obese (Table 1).

**Table 1:** Sociodemographic and health-related characteristics of the participants (*n* = 677).

Variable	Mean ± Standard deviation	Min–Max	
Age (Years)	20.79 ± 2.34	18.00–30.00	
Sleep Duration (Hours)	7.40 ± 1.19	4.00–13.00	
BMI (kg/m^2^)	23.18 ± 4.05	15.94–45.79	
		*n*	%
Sex	Female	509	75.2
	Male	168	24.8
Smoking status	User (Group 1)	140	20.7
	Non-user (Group 2)	537	79.3
Alcohol consumption	User (Group 1)	52	7.7
	Non-user (Group 2)	625	92.3
Physical activity	Active (Group 1)	185	27.3
	Inactive (Group 2)	492	72.7
Bristol stool type	Constipated	59	8.7
	Normal	578	85.4
	Diarrhea	40	5.9
BMI categories	Underweight	59	8.7
	Normal weight	439	64.8
	Overweight	140	20.7
	Obese	39	5.8

**Figure 1:**
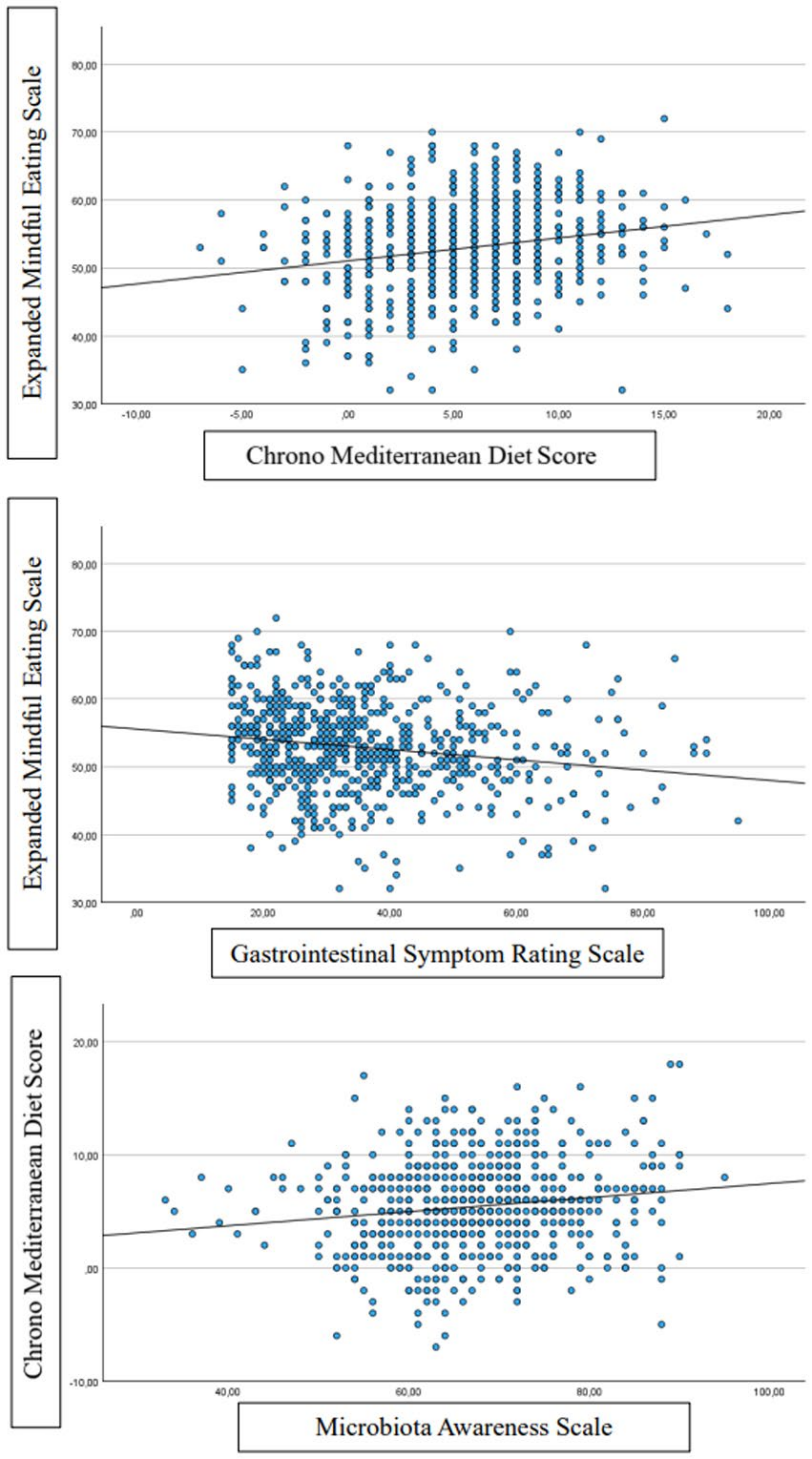
Scatterplots showing the relationships among the study scales.

Pearson correlation coefficients and descriptive statistics of the study variables are presented in Table 2. The mean GSRS score was 35.71 (SD = 15.66), the mean MAS score was 67.24 (SD = 9.53), the mean EMES score was 52.86 (SD = 6.53), and the mean CMDS score was 5.42 (SD = 3.94). The GSRS was negatively correlated with the MAS (*r* = −0.182, *p* < 0.001) and the CMDS (*r* = −0.141, *p* < 0.001). No significant correlation was found between the GSRS and the EMES (*r* = 0.047, *p* = 0.222). A positive correlation was observed between the MAS and the EMES (*r* = 0.092, *p =* 0.017). The CMDS was positively correlated with the MAS (*r* = 0.205, *p* < 0.001) and the EMES (*r* = 0.150, *p* < 0.001). The relationships between the variables are illustrated in Figure 1.

**Table 2:** Pearson correlation coefficients and descriptive statistics of the study scales (*n* = 677).

Scale	Median	Mean	SD	GSRS	MAS	EMES	CMDS
GSRS	32.00	35.71	15.66	—			
MAS	67.00	67.24	9.53	−0.182**	—		
EMES	53.00	52.86	6.53	0.047	0.092*	—	
CMDS	5.00	5.42	3.94	−0.141**	0.205**	0.150**	—

**p < 0.001; *p < 0.05. GSRS, Gastrointestinal Symptom Rating Scale; MAS, Microbiota Awareness Scale; EMES, Expanded Mindful Eating Scale; CMDS, Chrono-Mediterranean Diet Score.

A multiple linear regression analysis was conducted to examine the predictors of gastrointestinal symptoms as measured by the GSRS. Preliminary analyses were performed to assess the assumptions of the regression model. The Durbin–Watson statistic was 1.99 (*p* = 0.419). The overall regression model was statistically significant, *F*(14, 662) = 5.86, *p* < 0.001, explaining 11% of the variance in GSRS scores (*R*^2^ = 0.11, adjusted *R*^2^ = 0.09). The results of the regression analysis are presented in Table 3. Among the predictors, sex, smoking status, mindful eating, and Chrono-Mediterranean diet adherence were significantly associated with the GSRS scores. Male participants reported significantly lower GSRS scores than female participants (*β* = −0.21, *p* < 0.001). Participants who did not smoke reported significantly lower GSRS scores than those who smoked (β = −0.12, *p* = 0.005). Higher EMES scores were associated with lower GSRS scores (β = −0.16, *p* < 0.001). Similarly, higher CMDS scores were negatively associated with GSRS scores (β = −0.11, *p* = 0.007). Age, alcohol consumption, physical activity, sleep duration, microbiota awareness (MAS), BMI categories, and Bristol stool types (constipation and diarrhea) were not significantly associated with GSRS scores (*p* > 0.05) (Table 4).

**Table 3:** Linear regression analysis for variables predicting GSRS scores (*n* = 677).

Variable	*B*	*SE*	95.00% CI	β	*t*	*p*
(Intercept)	56.17	8.65	[39.18, 73.16]	0.00	6.49	< 0.001
Age	0.10	0.25	[−0.40, 0.59]	0.01	0.38	0.706
Sex (Male)	−7.70	1.47	[−10.58, −4.82]	−0.21	−5.25	< 0.001
Smoking (Non-user)	−4.65	1.64	[−7.86, −1.44]	−0.12	−2.84	0.005
Alcohol (Non-user)	−0.43	2.51	[−5.36, 4.50]	−0.007	−0.17	0.865
Physical Activity (Active)	1.15	1.36	[−1.52, 3.83]	0.03	0.85	0.397
Sleep Duration	−0.19	0.49	[−1.15, 0.77]	−0.01	−0.39	0.696
MAS Score	0.09	0.06	[−0.03, 0.21]	0.05	1.41	0.158
EMES Score	−0.37	0.09	[−0.55, −0.19]	−0.16	−4.07	< 0.001
Chrono-Mediterranean Diet Score	−0.43	0.16	[−0.74, −0.12]	−0.11	−2.71	0.007
Bristol (Constipated)	3.96	2.06	[−0.08, 8.00]	0.07	1.92	0.055
Bristol (Diarrhea)	4.75	2.47	[−0.10, 9.60]	0.07	1.92	0.055
BMI (Underweight)	−3.28	2.10	[−7.40, 0.84]	−0.06	−1.56	0.118
BMI (Overweight)	1.29	1.50	[−1.65, 4.23]	0.03	0.86	0.389
BMI (Obese)	1.95	2.56	[−3.07, 6.97]	0.03	0.76	0.445

*F*(14, 662) = 5.86, *p* < 0.001, *R*^2^ = 0.11, adjusted *R*^2^ = 0.09.

**Table 4:** Multiple linear regression analysis for variables predicting Chrono-Mediterranean diet score (*n* = 677).

Variable	B	SE	95% CI	*β*	*t*	*p*
(Intercept)	−5.11	2.12	[−9.27, −0.95]	0.00	−2.41	0.016
Age	−0.01	0.06	[−0.13, 0.11]	−0.007	−0.19	0.847
Sex (Male)	0.18	0.36	[−0.53, 0.89]	0.02	0.49	0.623
Smoking (Non-user)	0.82	0.40	[0.03, 1.61]	0.08	2.04	0.042
Alcohol (Non-user)	1.35	0.62	[0.14, 2.56]	0.09	2.19	0.029
Physical Activity (Active)	2.07	0.33	[1.44, 2.71]	0.23	6.38	<0.001
Sleep Duration	0.003	0.12	[−0.23, 0.24]	0.0009	0.02	0.981
MAS Score	0.05	0.02	[0.02, 0.08]	0.12	3.23	0.001
EMES Total Score	0.09	0.02	[0.05, 0.14]	0.16	4.28	<0.001
Bristol (Constipated)	−0.31	0.51	[−1.31, 0.68]	−0.02	−0.62	0.536
Bristol (Diarrhea)	−0.24	0.61	[−1.43, 0.95]	−0.01	−0.39	0.695
BMI (Underweight)	−0.68	0.52	[−1.69, 0.34]	−0.05	−1.31	0.190
BMI (Overweight)	0.49	0.37	[−0.24, 1.21]	0.05	1.32	0.187
BMI (Obese)	−1.26	0.63	[−2.49, −0.03]	−0.07	−2.01	0.045

*F*(13, 663) = 8.97, *p* < 0.001, *R*^2^ = 0.15, adjusted *R*^2^ = 0.13.

A multiple linear regression analysis was performed to identify the predictors of CMDS values. The Durbin–Watson statistic was 1.81, indicating no significant autocorrelation among the residuals. The overall regression model was statistically significant, *F*(13, 663) = 8.97, *p* < 0.001, explaining 15% of the variance in CMDS scores (*R*^2^ = 0.15, adjusted *R*^2^ = 0.13). Several variables were identified as significant predictors. Engaging in physical activity was the strongest predictor, with physically active individuals scoring on average 2.07 points higher than inactive participants (B = 2.07, *p* < 0.001). Abstinence from smoking (B = 0.82, *p* = 0.042) and from alcohol consumption (B = 1.35, *p* = 0.029) were also associated with higher CMDS scores. Higher microbiota awareness (MAS) scores were positively associated with CMDS scores (B = 0.05, *p* = 0.001). Similarly, higher EMES scores were positively associated with CMDS scores (B = 0.09, *p* < 0.001). Regarding body mass index, being in the obese category was a significant negative predictor, decreasing the mean CMDS score by 1.26 points (B = −1.26, *p* = 0.045). Age, sex, sleep duration, Bristol stool types (constipation and diarrhea), and underweight and overweight BMI categories were not significantly associated with CMDS scores (*p* > 0.05).

## 4 Discussion

This study provides evidence that gastrointestinal symptom burden in young adults may be associated with dietary patterns, mindful eating, and health-related behaviors within the context of the gut–brain interaction framework. The main findings indicate that greater adherence to the Chrono-Mediterranean diet and higher levels of mindful eating are associated with lower gastrointestinal symptom burden. Although microbiota awareness also correlated with symptom burden, it was not an independent predictor in the multivariate model. In addition, the co-occurrence of Chrono-Mediterranean diet adherence with physical activity, abstinence from smoking and alcohol consumption, and higher levels of mindful eating suggests that gastrointestinal symptoms should be interpreted within a broader network of interrelated lifestyle and behavioral factors rather than as isolated outcomes. Collectively, these findings may support the view that symptom burden in gut–brain interactions may be shaped not only by biological mechanisms but also by behavioral and diet-related components. Given the cross-sectional nature of this study, these findings indicate associations rather than causal relationships, and the direction of these relationships cannot be determined. It is also possible that individuals experiencing greater gastrointestinal symptoms may modify their dietary habits or eating behaviors, indicating potential reverse associations that may partially influence the observed relationships. Although the regression model predicting gastrointestinal symptom burden was statistically significant, it explained a relatively modest proportion of the variance (*R*^2^ = 0.11), suggesting that gastrointestinal symptoms in young adults are likely to be influenced by additional factors beyond those captured in the present study. In terms of relative contribution, standardized coefficients indicated that male sex, higher eating awareness, non-smoking, and greater adherence to the Chrono-Mediterranean diet were the main independent correlates of lower symptom burden. Although sleep duration and physical activity were included in the model, they were not significantly associated with GSRS scores in this sample. Nevertheless, other potentially relevant factors, particularly stress, anxiety, broader psychosocial burden, and additional unmeasured lifestyle characteristics, may also contribute to gastrointestinal symptoms and should be explored in future studies using more comprehensive models.

This study demonstrated that the burden of gastrointestinal symptoms in young adults is particularly associated with dietary patterns and mindful eating. The most notable finding is that higher CMDS and higher levels of mindful eating are associated with a lower burden of gastrointestinal (GI) symptoms. Our finding that a higher CMDS score was associated with a lower gastrointestinal symptom burden is broadly consistent with a growing body of literature suggesting that Mediterranean-type dietary patterns may be beneficial for functional gastrointestinal health. In a survey from Southern Italy, Zito et al. reported that individuals with irritable bowel syndrome (IBS) and functional dyspepsia had significantly lower adherence to the Mediterranean diet than controls, with this association being particularly evident in younger adults. Given that our study also focused on young adults, this parallel further suggests the potential relevance of Mediterranean-related dietary patterns in the gastrointestinal symptom burden in this age group (36). Similarly, Agakidis et al. showed that lower adherence to the Mediterranean diet was associated with a higher prevalence of functional gastrointestinal disorders in children and adolescents; notably, adherence to the Mediterranean diet remained a significant predictor even after adjusting for age subgroups. Although their study involved a younger population, consistency with our findings suggests that the relevance of Mediterranean-type dietary patterns for gastrointestinal health extends across different age groups (37). This interpretation is also supported by broader evidence indicating that overall dietary pattern quality matters for gastrointestinal outcomes. In a large prospective cohort, Wu et al. found that higher consumption of ultra-processed foods was associated with a significantly greater risk of incident IBS in a dose–response manner. Although this study did not directly assess Mediterranean diet adherence, it reinforces the broader notion that gastrointestinal outcomes may be shaped by overall diet quality rather than by isolated dietary components alone (38). Similarly, a recent systematic review by Ashoorion et al. reported that higher adherence to the Mediterranean diet was associated with a lower likelihood of functional dyspepsia in adults. At the same time, that review also suggested that not all generally healthy dietary patterns are equally beneficial for gastrointestinal outcomes because certain Dietary Approaches to Stop Hypertension (DASH)-based patterns were associated with increased postprandial distress syndrome. Taken together, these findings indicate that gastrointestinal symptom burden may depend not only on general diet quality but also on the specific composition and structure of the dietary pattern (39). However, the available literature also suggests that the association between Mediterranean-type dietary patterns and gastrointestinal symptoms is not uniform across all settings. Chen et al. found that standard Mediterranean diet adherence was not associated with IBS symptom severity, abdominal pain, or bloating in patients with IBS, suggesting that the relationship may be more heterogeneous in specific clinical populations and may be influenced by individual symptom triggers, food composition, and the need for dietary personalization (40). Nevertheless, emerging interventional evidence provides additional support for the potential beneficial role of Mediterranean dietary patterns. In a 6-week randomized controlled trial, Staudacher et al. reported that Mediterranean diet counseling improved gastrointestinal symptoms and depressive symptom response in adults with IBS compared with a habitual diet (41). Together, these findings suggest that Mediterranean-type dietary patterns may be associated with lower gastrointestinal symptom burden at the population level, while their effects in specific clinical conditions may vary depending on the symptom phenotype and individual tolerance.

Mechanistic evidence also helps contextualize our findings. Barber et al. demonstrated that a fiber-enriched Mediterranean-type diet altered microbial metabolic pathways, increased the abundance of specific butyrate-producing taxa, and modified digestive sensations in healthy adults, even without significant changes in overall microbiota composition. These findings suggest that Mediterranean-related dietary patterns may be linked to gastrointestinal symptom burden through functional changes in the gut environment and microbial metabolism, rather than solely through structural shifts in microbiota composition (42). The same study also reported increased flatulence and borborygmi, likely reflecting enhanced fermentative activity and greater colonic biomass rather than an adverse gastrointestinal pathology. Similarly, Mitsou et al. found that higher adherence to the Mediterranean diet was associated with several potentially favorable gut microbiota characteristics, including lower *Escherichia coli* counts and a higher *Bifidobacterium*: *E. coli* ratio, but also with more pronounced gastrointestinal symptoms and higher defecation frequency (43). These observations suggest that Mediterranean-type dietary patterns may not uniformly reduce all gastrointestinal sensations and that some symptom changes may reflect altered fermentation, bowel activity, or gastrointestinal physiology rather than an unfavorable clinical response. An additional point that is particularly important in interpreting our findings is the nature of the dietary assessment tools used in this study. Mediterranean diet adherence was evaluated using the CMDS; the Turkish version has recently been shown to be a valid and reliable instrument for adult populations (21). Unlike conventional Mediterranean diet scores, the CMDS incorporates not only food group-based adherence but also the timing of farinaceous product intake and physical activity. This broader framework may allow for the assessment of more integrated dietary and lifestyle patterns. Therefore, the inverse association observed between CMDS and gastrointestinal symptom burden in our study may reflect not only Mediterranean-type food choices but also chrono-nutritional and behavioral components that are relevant to gut–brain interaction. Overall, our findings align with the evidence from a broader review suggesting that Mediterranean-type dietary patterns may play a protective role in functional gastrointestinal disorders. As summarized by Cenni et al., greater adherence to the Mediterranean diet has been associated with a lower prevalence of functional gastrointestinal disorders (FGIDs) and more favorable symptom outcomes, although the authors also emphasized the need for larger and longer-term studies (44). Within this broader framework, our results suggest that adherence to Chrono-Mediterranean dietary patterns is associated with a lower gastrointestinal symptom burden in young adults.

The lower gastrointestinal symptom burden observed in men suggests sex-related differences in the epidemiology and presentation of gut–brain interaction disorders in young adults. This interpretation is broadly consistent with previous evidence that women tend to report a higher prevalence and greater symptom burden of several functional gastrointestinal symptoms and related conditions. Possible explanations include hormonal influences on visceral sensitivity, gastrointestinal motility, intestinal barrier function, and neuroimmune signaling, together with psychosocial factors, differences in stress response, and sex-related variation in symptom reporting (45–47). Moreover, studies on IBS and related disorders have shown that women may experience poorer psychosocial outcomes, including higher levels of fatigue, anxiety, and depression, and a lower quality of life, which may contribute to the greater symptom burden reported in female participants (47).

The finding that non-smoking was associated with a lower gastrointestinal symptom burden is consistent with previous evidence suggesting that smoking may adversely affect a range of functional gastrointestinal symptom profiles. In three population-based studies, Talley et al. reported that cigarette smoking was independently associated with higher odds of postprandial distress syndrome, whereas heavy smoking was associated with IBS-diarrhoea (diarrhoea-predominant IBS), diarrhoea as a symptom, urgency to defecate, and flatulence (48). Similarly, Lundström et al. reported associations between smoking and functional abdominal pain, bloating, and constipation. In addition, a Turkish study conducted among treatment-seeking smokers found that gastrointestinal symptoms decreased after smoking cessation in individuals with IBS, although the IBS status itself did not change significantly (49). Overall, these findings support the possibility that smoking may be associated with a broader burden of gastrointestinal symptoms rather than with isolated symptom categories (48, 50).

Although mindful eating was not significantly correlated with gastrointestinal symptom burden in the univariate analysis, its independent association in the multivariable model is noteworthy. This finding suggests that the relevance of mindful eating becomes more apparent after accounting for other lifestyle and individual factors, which may obscure its effect at the bivariate level. From a behavioral perspective, mindful eating may be linked to gastrointestinal symptoms through greater sensitivity to hunger and satiety cues, slower and more deliberate eating, better recognition of symptom-triggering foods, and reduced emotionally driven eating. In this context, Naliboff et al. showed that improvement in the “act with awareness” facet of mindfulness was the strongest predictor of gastrointestinal symptom improvement in individuals with IBS, suggesting that present-moment attention and more intentional behavioral regulation may be important for gastrointestinal outcomes (51). Similarly, Sinn et al. reported that rapid eating was associated with functional dyspepsia in young women, indicating that the manner in which food is consumed, and not only what is consumed, may be relevant to symptom experience (52). In addition, Jia et al. found that abnormal eating styles were associated with higher odds of IBS, highlighting the possible role of psychologically driven eating behaviors in gastrointestinal symptom presentation (53). Taken together, these findings suggest that the inverse association observed between mindful eating and gastrointestinal symptom burden in our study may reflect the potential relevance of a more adaptive and attentive eating pattern, although this relationship is not evident in simple correlation analyses (51–53). An alternative explanation is that individuals with higher health consciousness may simultaneously adopt multiple health-promoting behaviors, which could partially account for the observed associations without implying a unified lifestyle pattern.

Although microbiota awareness was found to be correlated with lower gastrointestinal symptom burden and higher CMDS scores, it was not an independent predictor in the multivariable model. This suggests that microbiota awareness functions more as an indirect correlate of gastrointestinal health than as a direct determinant of symptom burden. In other words, awareness alone may not be sufficient to influence clinical symptoms unless it is translated into sustained dietary and lifestyle behaviors. This interpretation is supported by previous studies that have shown a gap between microbiota-related knowledge and actual practice. For example, among Jordanian athletes, familiarity with probiotics and prebiotics was not accompanied by a higher intake of gut-supportive foods or significant differences in gastrointestinal symptoms (54). Similarly, a large knowledge–attitude–practice survey of Chinese parents showed that although knowledge regarding gut microbiota was positively related to attitudes and practices, substantial gaps and misunderstandings remained (55). Similarly, Ahmed et al. reported that, although the majority of participants were aware of the diet–stress–microbiota connection, daily intake of probiotic- and fiber-rich foods remained low, and gastrointestinal complaints were still common (56). Taken together, these findings suggest that microbiota-related awareness may reflect health-oriented intentions, but its clinical relevance depends on whether it results in actual and sustained behavioral changes. This interpretation is also consistent with the cross-sectional nature of our study and the absence of direct microbiota measurements or longitudinal dietary data, both of which may have limited our ability to detect the longer behavioral and biological pathways linking awareness to symptom outcomes. If we had had access to longitudinal dietary records, microbiota-related biomarkers, or direct gut microbiome analyses, we might have been able to more clearly define the contribution of microbiota awareness.

The predictor profile of CMDS in our study suggests that adherence to Chrono patterns may extend beyond dietary intake alone and may be associated with broader healthy lifestyle patterns, although this cannot be fully established due to the cross-sectional design. In particular, the strong positive association with physical activity, together with positive associations with not smoking or drinking alcohol, mindful eating, and microbiota awareness, indicates that higher CMDS values are associated with a cluster of other health-promoting behaviors. This interpretation is consistent with the Mediterranean lifestyle framework, which conceptualizes adherence to the Mediterranean diet as a multidimensional construct that includes not only dietary habits but also physical activity, rest, and other lifestyle behaviors (57). Our findings are also in line with those of previous studies showing that adherence to Mediterranean dietary patterns is embedded in a broader lifestyle profile. In adults, Patino-Alonso et al. found that greater physical activity was associated with better Mediterranean diet adherence, whereas obesity and male sex were linked to poorer adherence (58). Similarly, in a university student population, Castro-Cuesta et al. reported that physically active students had higher adherence to the Mediterranean diet, whereas sedentary students were more likely to show low adherence, which is particularly relevant given that our sample also consisted of young adults (59). Supporting this broader interpretation, Bennasar-Veny et al. further showed that Mediterranean diet adherence in university students was positively associated with physical activity and negatively associated with tobacco consumption, and their cluster analysis identified distinct healthy and unhealthy lifestyle patterns (60). Additional evidence comes from Lara et al., who evaluated Mediterranean diet adherence together with smoking, physical activity, and BMI within a wider health behavior score, and also support the view that Mediterranean-type dietary patterns may cluster with other health-related behaviors rather than operate in isolation (61). The inverse association between CMDS and obesity observed in our study further reinforces this interpretation. This finding is in line with synthesis-level evidence showing that greater adherence to the Mediterranean diet is associated with a lower risk of overweight/obesity and with less long-term weight gain, suggesting that greater adherence to the Chrono-Mediterranean diet may be linked to a more favorable behavioral and metabolic profile (62). This broader interpretation may be particularly relevant given the chrono-nutritional structure of CMDS. Muscogiuri et al. reported that lower adherence to the Mediterranean diet was associated with an evening chronotype, lower physical activity, higher prevalence of smoking, and a higher BMI, suggesting that adherence to the Mediterranean diet may also be intertwined with circadian and lifestyle-related characteristics (63). Taken together, these findings suggest that adherence to Chrono-Mediterranean patterns is shaped not only by food choices but also by a wider constellation of health behaviors and lifestyle characteristics.

In conclusion, this study demonstrates that greater adherence to the Chrono-Mediterranean diet and higher levels of mindful eating in young adults are associated with a lower burden of gastrointestinal symptoms. Microbiota awareness, on the other hand, appears to be more closely linked to healthy behavioral patterns. The findings suggest the potential relevance of nutrition- and mindfulness-based approaches in relation to gastrointestinal symptoms within the framework of gut–brain interaction.

### 4.1 Strengths, limitations, and future directions

The main strengths of this study include its relatively large sample size, focus on young adults, and multidimensional evaluation of gastrointestinal symptom burden in relation to dietary patterns, mindful eating, microbiota awareness, and lifestyle factors. In addition, the combined use of correlation and multiple regression analyses allowed for a more comprehensive assessment of the relationships between the study variables.

Several methodological limitations should be considered when interpreting these findings. First, the cross-sectional design precludes causal inferences; therefore, the observed associations should not be interpreted as directional or causal relationships. Second, reliance on self-reported measures may have introduced recall bias and affected data accuracy. Third, the use of convenience and snowball sampling introduces a potential risk of selection bias, as individuals with a greater interest in health-related topics may have been more likely to participate, which may have influenced the observed associations. In addition, the disproportionately high proportion of female participants limits the generalizability of the findings, particularly in male populations. Finally, the study assessed awareness of the gut microbiota rather than directly measuring its composition and therefore reflects perceived rather than objective microbiota-related characteristics. Future research may benefit from longitudinal or prospective designs to clarify the temporal relationships between gastrointestinal symptom burden, dietary patterns, mindful eating, and microbiota awareness. Studies with more diverse and sex-balanced samples would improve the generalizability of the findings. Moreover, incorporating objective or complementary measures, such as repeated 24-h dietary recalls, multi-day food records, meal timing assessments, clinical evaluations of gastrointestinal symptoms, and direct characterization of gut microbiota composition, may provide a more complete understanding of the observed associations. Intervention studies could also help determine whether improvements in Chrono-Mediterranean diet adherence and mindful eating are accompanied by meaningful changes in gastrointestinal symptom burden in young adults.

## 5 Conclusion

Greater adherence to the Chrono-Mediterranean diet and higher levels of mindful eating were independently associated with a lower gastrointestinal symptom burden in young adults. Although microbiota awareness was associated with symptoms in the univariate analyses, it was not an independent predictor. Adherence to the Chrono-Mediterranean diet also clustered with other health-promoting behaviors, suggesting that it may reflect a broader lifestyle pattern. These findings underscore the potential role of modifiable dietary and behavioral factors in the experience of gastrointestinal symptoms and may be relevant within the framework of disorders involving gut–brain interaction. However, further longitudinal studies are required to confirm this association.

## Data Availability

The raw data supporting the conclusions of this article will be made available by the authors, without undue reservation. Requests to access these datasets should be directed to canan.altinsoy@erdogan.edu.tr or isa.celik@erdogan.edu.tr.
